# Pre-analytical and analytical procedures to avoid loss of vitamin C. Comment on: “COVID-19: up to 82% critically ill patients had low vitamin C values”

**DOI:** 10.1186/s12937-022-00803-y

**Published:** 2022-07-29

**Authors:** Luis Chiscano-Camón, Juan Carlos Ruiz-Rodriguez, Roser Ferrer, Sílvia Camós, Adolf Ruiz-Sanmartin, Ricard Ferrer

**Affiliations:** 1 Intensive Care Department, Vall d’Hebron Hospital Universitari, Vall d’Hebron Barcelona Hospital Campus, Barcelona, Spain; 2 Shock, Organ Dysfunction and Resuscitation Research Group, Vall d’Hebron Institut de Recerca (VHIR), Vall d’Hebron Hospital Universitari, Vall d’Hebron Barcelona Hospital Campus, Barcelona, Spain; 3 Departament de Medicina, Universitat Autònoma de Barcelona, Bellaterra, Spain; 4 Biochemistry Department, Clinical Laboratories, Vall d’Hebron University Hospital, Vall d’Hebron Barcelona Hospital Campus, Barcelona, Spain

## Main text

We have read with great interest the short report by Tomasa – Irriguible et al. about the vitamin C levels in critically ill COVID-19 adult patients with ARDS [1]. It refers to our study, in which it was found that in patients with ARDS caused by COVID-19, vitamin C levels were extremely low [2]. However, the results of our study are questioned on the basis that according to Tomasa – Irriguible et al., the veracity of the methodology used to assess the vitamin C status of the patients was not clear, so the values could be artifactually low.

According to the literature, vitamin C is a very unstable water-soluble micronutrient which can be easily oxidized or hydrolyzed [3]. We agree with the authors that the determination of vitamin C levels requires very rigorous pre-analytical and analytical procedures to avoid loss of vitamin. In our study we followed the recommendations published by Pullar et al. [4] and we performed the pre-analytical phase with great care.

In our case, venous blood was collected into a 4 mL vacuette tube with anticoagulant lithium heparin and immediately delivered to the laboratory protected from light. Two aliquots of plasma were separated in a centrifuge at 2.643 g for 10 min and stored frozen at − 20 °C protected from light until their analysis (within 7 days of arrival - stability of the vitamin C molecule is 7 days at − 15 °C).

To extract and stabilize vitamin C an equal volume of cold metaphosphoric acid was added to the samples while kept on ice bath and protected from light exposure. Plasma vitamin C was analyzed by a reversed-phase high-performance liquid chromatography (HPLC) method with photodiode detector (wavelength set to 245 nm). The method was fully validated in terms of linearity (1.5–30 mg/L), imprecision (less than 5%) and accuracy [5].

The differences observed between the series by Tomasa-Irriguible et al. and ours can be explained by three reasons. First of all, the timing of the sample collection; thus we consider this the most important consideration regarding the difference in the results. While they studied vitamin C in the first 24 hours after ICU admission, our study reported vitamin C serum levels at 17.5 ± 1.7 days from admission to ICU. Second, the levels considered low by Tomasa (0.14 ± 0.05 mg / dL) would have been considered undetectable by the stoppage limit of our laboratory. So if their patients had been studied by our detection threshold, there would be 65 patients (82%) who would present undetectable levels. Third, our patients had a higher degree of lung inflammation. In our series, 94.4% of patients required prone position with P_a_O_2_/F_i_O_2_ ratio at the time of vitamin C measurement of 94.4 ± 5.9, unlike the series with which we compared ourselves, where only 49 (73.1%) required mechanical ventilation.

We consider that our preanalytical processing of the samples for the analysis of vitamin C was carried out under conditions that guarantee the absence of oxidation of vitamin C and stability of the sample throughout the whole process. The method is robust for clinical measurement of vitamin C in plasma specimens. Consequently, the results of our study should be considered correct.

## Data Availability

The datasets used and/or analysed during the current study are available from the corresponding author on reasonable request.

## Abbreviations

ARDS: Acute Respiratory Distress Syndrome
COVID-19: Coronavirus Infectious Disease 2019
ICU: Intensive Care Unit
ml: Milliliters
PaO2/FiO2 ratio: Arterial oxygen pressure (PaO2), inspired fraction of oxygen (FIO2)
SARS-CoV-2: Severe acute respiratory syndrome Coronavirus-2
